# CD226 deficiency improves cognitive functions and ameliorates anxiety‐like behaviors in mice

**DOI:** 10.1002/brb3.871

**Published:** 2017-11-22

**Authors:** Liang Fang, Jingyi Jin, Ping Chen, Ning Wang, Hanyu Zeng, Boquan Jin, Hui Li, Lihua Chen

**Affiliations:** ^1^ Department of Immunology The Fourth Military Medical University Xi'an China; ^2^ Department of Anatomy K.K. Leung Brain Research Centre The Fourth Military Medical University Xi'an China

**Keywords:** anxiety, CD226, memory

## Abstract

**Background:**

CD226 is a cell surface adhesion molecule expressed in the immune system and central nervous system. Although the role of CD226 in the function of immune cells has been well studied, there has been no report on the potential functional significance of CD226 in neural cells.

**Methods:**

We investigated the role of CD226 on the cognitive function and behaviors using CD226 knockout (CD226KO) and wild‐type mice. The spatial learning and memory were characterized using Morris water maze test, and the behaviors were evaluated using open field and elevated plus maze tests. IL‐10 expression in the hippocampus was measured using RT‐PCR and ELISA.

**Results:**

The results showed that CD226KO mice displayed increased spatial learning and memory than the wild‐type controls. We also found that genetic deletion of CD226 resulted in decreased anxiety‐like behaviors. In addition, the hippocampal expression level of IL‐10 was increased in the CD226KO mice compared with the WT mice.

**Conclusions:**

Our findings suggest that CD226 plays an important role in the modulation of cognition and anxiety in mice.

## 1 INTRODUCTION

CD226, also known as PTA1, TliSA1, or DNAM‐1, is a cell surface adhesion molecule found on most immune cells including T cells, a subset of B cells, natural killer (NK) cells, natural killer T (NKT) cells, monocytes, and platelets in both humans and mice (He & Tian, 2016; Sherrington et al., 1997; Shibuya et al., 1996; Tahara‐Hanaoka et al., 2005; Vo, Takenaka, Shibuya, & Shibuya, 2016; Zeng, Zhang, Jin, & Chen, 2015). CD226 functions as a costimulatory molecule by interacting with its ligands (CD155 and CD112), and is involved in various immunological functions including T‐cell differentiation, NK cell cytotoxicity, monocyte extravasation, and dendrite cell maturation (Ayano et al., 2015; Bottino et al., 2003; Gilfillan et al., 2008; Iguchi‐Manaka et al., 2008; Nabekura et al., 2010; Tahara‐Hanaoka et al., 2005).

Cell adhesion molecules (CAMs) maintain the physical contact between opposing membranes. To date, accumulating studies indicate that the CAMs play a key role in synaptic plasticity, including synapse specification, initial target recognition, and synapse maturation during emotion, learning, and memory processes (Mizoguchi et al., 2002; Sudhof, 2008; Yamagata, Sanes, & Weiner, 2003). As a member of CAMs, CD226 possesses the typical structure of the other identified synapse‐related CAMs, that is, single‐spanning membrane proteins with several Ig‐like extracellular domains and an intracellular tail containing a PDZ‐binding motif characterized by a conserved sequence (Sherrington et al., 1997). In addition, our previous study found CD226 was located in the mouse hippocampus and cerebellum. It was co‐localized well with synaptic marker proteins including syntaxin, synaptophysin, and PSD‐95 (Zhang et al., 2009). These finding implied that CD226 may play key roles in the synaptogenesis.

To date, the role of CD226 on cognition and behaviors has not been defined. In view of the above studies, we hypothesized that brain CD226 is involved in mediating synaptic plasticity. We therefore examined the role of CD226 by comparing cognitive function and behaviors between CD226 knockout (KO) mice and wild‐type (WT) controls. Three classic rodent behavioral tests including the Morris water maze (MWM) test, open field (OF) test, and the elevated plus maze (EPM) test were utilized to analyze the behavioral performance of mice.

## 2 MATERIALS AND METHODS

### 2.1 Animals

Mice with homozygous deletions of CD226 genes (*CD226* ^−/−^) on a C57BL/6 background were kindly provided by Professor Marco Colonna. C57BL/6 mice (8 weeks) were purchased from Yison BIO (Shanghai, China). CD226^−/−^ mice were back‐crossed to C57BL/6, then propagated by CD226^+/−^ × CD226^+/−^ mating. WT mice (CD226^+/+^) used as control in our experiments are the littermates of *CD226* ^−/−^ mice. Mice were maintained in a specific pathogen‐free room at Experimental Animals Center of Fourth Military Medical University. The experimental procedures were approved by the Institutional Review Board (IRB) of the Fourth Military Medical University (permit number XJYYLL‐2014433). All animals were treated according to the Guide for the Care and Use of Laboratory Animals (NIH, Bethesda, MD).

### 2.2 Morris water maze test

The Morris Water Maze test (MWM) was conducted to evaluate visual‐spatial learning. The swimming activity of each mouse was recorded using a camera mounted overhead. A video‐tracking system (Ethovision 3.0, Noldus Information Technology, Netherlands) was used to collect the movement data (escape latency, swim path, distance, and speed). In hidden platform test, each animal was trained four times per day for five consecutive days. Then, each animal was given a 120 s probe trial to evaluate its ability of memory retention on day 6.

### 2.3 Open field test

Overall locomotive activity and anxious behaviors were evaluated in the open field (OF) test. The field consisted of a square black plexiglass box (30 cm long × 30 cm wide × 25 cm high), with an outlined center area (15 cm long × 15 cm wide). The mice were individually placed into the box for 10 min. At the beginning of each test, every animal was introduced to the same corner and were given 1 min to get used to the experimental environment. A computerized video‐tracking system (Ethovision XT, Noldus Information Technology, Netherlands) recorded the data. Several parameters were recorded, including the total path length, path length in the center, path length in the periphery surrounding the center (i.e., total area minus center area), and path length in the corners (7 cm × 7 cm), as well as the number of entries and time spent in the different areas of the open field. The center time in the first 5 min reflects the state of anxiety in the strange new environment; the total distance that was traveled by the mice in 30 min reflected the locomotive activity of the mice.

### 2.4 Elevated plus maze test (EPM)

The elevated plus maze consisted of two open arms (30 cm × 5 cm) and two opposite arms (30 cm × 5 cm) enclosed by high walls (15 cm) that extended from a central platform (5 cm^2^). The maze was placed approximately 38 cm above the floor. At the beginning of a test, each mouse was placed facing the same closed arm and released to explore the maze for 5 min freely. The total time spent in the open versus the closed arms was recorded. As mice are naturally afraid of open space, the anxiolytic effect was defined by an increase in the time spent in the open arms.

### 2.5 Quantitative real‐time PCR

Total RNA was extracted from hippocampus tissue using the Trizol reagent (Invitrogen, Carlsbad, CA USA). cDNA was synthesized using PrimeScript™ RT Master Mix (Takara). Real‐time PCR was performed on 7500 cycler (Applied Biosystems, Forest City, CA) according to the manufacturer's instructions using SYBR® Premix Ex Taq™ II (Takara). PCR thermal cycling conditions were 3 min at 95°C, and 40 cycles of 15 s at 95°C, 58 s at 60°C, 20 s at 72°C, and finally by extension at 72 °C for 5 min. Samples were run in triplicate. The PCR primers used for IL‐10 were 5′‐ TGGACAACATACTGCTAACCGAC ‐3′ and 5′‐CCACTGCCTTGCTCTTATTTTCAC‐3′; for GAPDH, were 5′‐GACAAAATGGTGAAGGTCGGT‐3′ and 5′‐GAGGTCAATGAAGGGGTCG‐3′. Expression of genes was determined with the ΔΔCt method. Relative expression levels of genes was calculated and normalized to GAPDH.

### 2.6 Measurement of IL‐10 production in tissue and serum

Immediately after behavioral testing, mice were killed by CO_2_ inhalation and decapitation. The hippocampus was quickly dissected from the brain and stored at −80° C. Serum of mice was also collected from eye ball venous blood. Plenty of IL‐10 was detected by ELISA assay (eBioscience) according to the manufacturer's instructions.

### 2.7 Statistical analysis

All data were presented as means ± SEM and were processed by SPSS 20.0 for Windows. Statistical analysis was carried out using two‐way repeated measures ANOVA tests and Mann–Whitney rank‐sum test for MWM test data, *t* tests for OF test and EPM test data. *p *<* *.05 was considered significant.

## 3 RESULTS

### 3.1 CD226 deficiency enhanced the spatial learning and memory of mice

**Figure 1:**
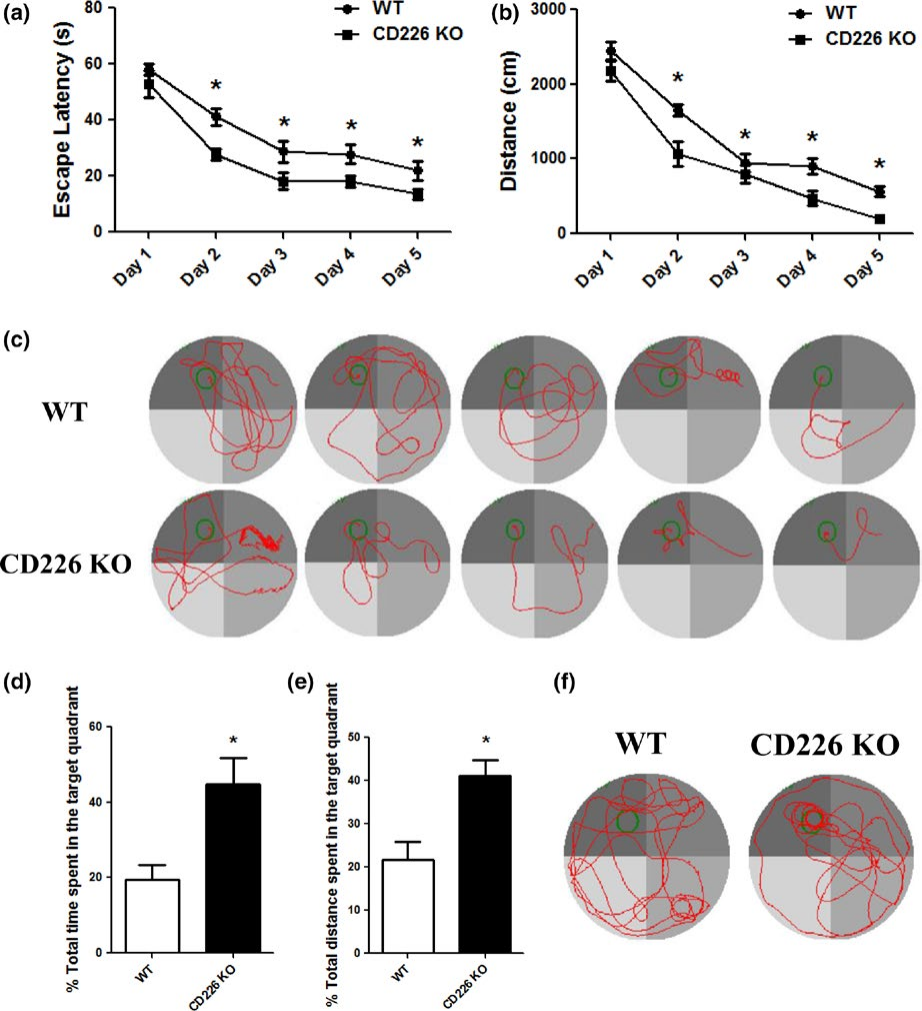
CD226 deficiency enhanced spatial learning and memory in mice in Morris water maze test. (a and b) Graph showing the average escape latencies and distances of mice in searching for the hidden platform during five consecutive training days. (*n* = 10, *p <* *.05 compared with WT control). (c) Representative swimming traces of mice in two groups during the hidden platform test. The large circle represents the water maze pool and the small circle represents the platform. (d and e) Histograms showing the increase in swimming time and distance in the target quadrant during probe trial test. (*n* = 10, *p <* *.05 compared with WT control). (f) Representative swimming traces during the probe test

*CD226* ^−/−^ mice were used to study the effects of CD226 on the spatial learning and memory formation in mice. The memory and cognitive performance was assessed by five consecutive days of the Morris water maze test. As expected, during the five learning days, the mean escape latency and distance for the trained mice to find the hidden platform gradually decreased, indicating a progress in the learning task. We noticed that there was no difference in the escape latency and distance between WT and CD226KO mice on day 1, but CD226KO mice took less time and distance to arrive the hidden platform than did the WT mice on day 2–5 (Figure 1a, b, and c).

The ability of spatial memory was tested on day 6 by probe trials without platform, the percentage of swimming time spent in the target quadrant by CD226KO mice was increased compared to those of the WT mice (Figure 1d). Moreover, the percentage of total distance swum in the target quadrant was also increased in CD226KO mice compared with that of the WT group (Figure 1e and f). These results indicated that CD226 deletion induced the enhancement of spatial memory.

### 3.2 CD226 deficiency decreased the anxiety of mice in the open field test

**Figure 2:**
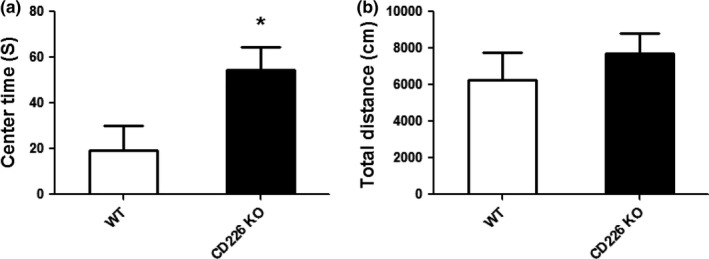
The effects of CD226 deficiency on anxiety‐like behaviors of mice in open field test. (a) Total time spent in the center of the open field. (b) Total distance travelled in the open field (*n* = 10, *p <* *.05 compared with WT control)

The anxiety‐like behaviors of CD226KO mice was evaluated in the open field test. Anxiety‐related behaviors were defined as decreased percentages of time spent in the center area. CD226KO mice exhibited lower anxiety‐like behaviors, as shown by increased time in the center region (Figure 2a). This decreased anxiety‐like behaviors of CD226 KO mice was not an artifact of reduced locomotor activity, as knockout of CD226 had no effect on total distance traveled (Figure 2b).

### 3.3 CD226 deficiency resulted in a specific decrease in anxiety‐related behaviors in the elevated plus maze test

**Figure 3:**
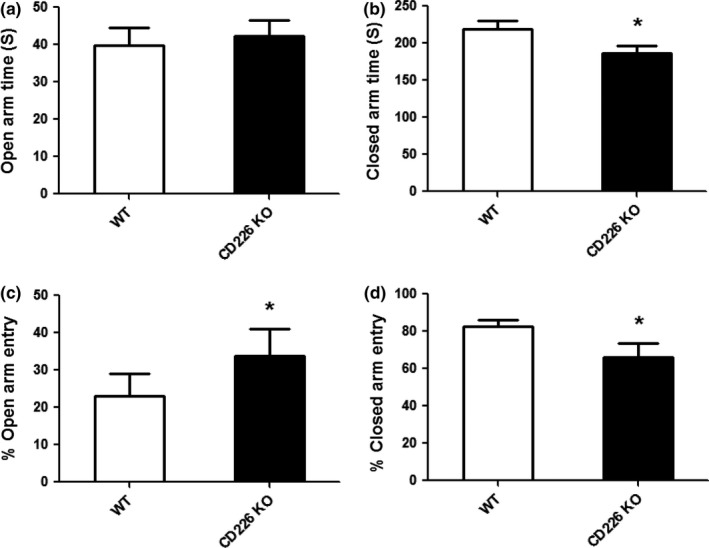
The effects of CD226 deficiency on anxiety‐like behaviors of mice in the elevated plus maze test. (a) Total time spent in the open arm of the elevated plus maze. (b) Total time spent in the closed arm of the elevated plus maze. (c) Total number of entries in the open arm of the maze. (d) Total number of entries in the closed arm of the maze (*n* = 10, *p <* *.05 compared with WT control)

To further confirm the role of CD226 in the anxiety of mice, exploratory activity of CD226KO mice and WT controls was assessed in the elevated plus maze (EPM). There was no difference in the total time spent in the open arms of the maze (Figure 3a), but the total time spent in the closed arms of the maze was significantly reduced in CD226KO mice compared to that in WT controls (Figure 3b). Moreover, the percentage of open arm entry was significantly increased in CD226KO mice compared to that in WT controls (Figure 3c), and the percentage of closed arm entry was reduced (Figure 3d). These observations suggest decreased anxiety in CD226KO mice.

### 3.4 CD226 deficiency increased IL‐10 expression in the hippocampus

**Figure 4:**
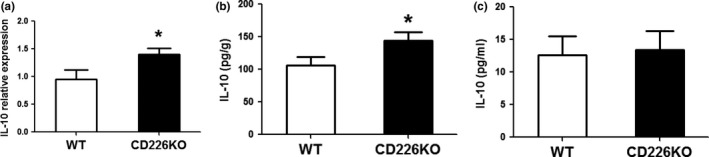
The effects of CD226 deficiency on IL‐10 expression in the hippocampus. (a) IL‐10 mRNA expression level in the hippocampus was determined. (*n* = 5, *p <* *.05 compared with that of WT control). (b) IL‐10 protein expression level in the hippocampus tissue homogenates was determined using ELISA. (*n* = 5, *p <* *.05 compared with that of WT control). (c) Serum IL‐10 expression level was determined

Interleukin (IL)‐10 is a prototypical anti‐inflammatory cytokine that may participate in behavior changes. As blockage of CD226 effectively increased the IL‐10 production in immune cells, we investigated whether IL‐10 was involved in CD226 deficiency‐mediated cognitive enhancement. We examined IL‐10 expression and found that IL‐10 mRNA expression level was increased in hippocampus of CD226KO animals (Figure 4a). Meanwhile, the supernatants of hippocampus tissue homogenates were used for the analysis of IL‐10. We found significantly higher level of IL‐10 in the tissue homogenate supernatants of CD226KO animals compared with that of WT controls (Figure 4b). However, serum IL‐10 did not differ between CD226KO and WT controls (Figure 4c).

## 4 DISCUSSION

Learning, memory, and anxiety are critical phenomena in central nervous system. Studies in both human patients and experimental rodents suggest that anxiety is often associated with reduced learning and memory. However, many questions remain unclear in the molecular underpinning of these behaviors. In this study, CD226 cognitive function was observed in the Morris water maze test measuring spatial learning and memory. A decrease in the time and distance to reach the hidden platform was observed in CD226KO mice during 5‐day learning compared with that of WT controls. The animals of CD226KO group also better remembered the location of the platform since they spent a lesser time in the target zone of the Morris water maze apparatus. These results implied that genetic deletion of CD226 selectively enhanced the hippocampus‐dependent spatial memory of mice. Moreover, the most robust behavioral indication for CD226 effect was observed in the OF test measuring anxiety‐like behaviors. CD226KO mice displayed decreased anxiety‐like behaviors, and this was further confirmed in EPM test. Furthermore, the analysis with cytokine expression in the hippocampus suggested that IL10 might be involved in CD226 deficiency‐mediated behavioral changes. This is the first study that associates CD226 with cognitive and emotional behaviors.

Cell adhesion molecules (CAMs) are mainly membrane glycoproteins that mediate cell–cell adhesion. Neural CAMs comprise the cadherin, integrin, and immunoglobulin superfamily (IgSF) that have been demonstrated to be involved in target‐cell recognition, neuronal cell migration, formation of complex glial networks, axon‐bundle formation, and activity‐dependent plasticity of synapses, which surround axons and synapses (Redies & Takeichi, 1996). CD226, an adhesion molecule belonging to the immunoglobulin superfamily, has well‐established roles in lymphocyte signaling, intercellular adhesion, cytotoxicity induced by T lymphocytes and NK cells, and cytokine secretion (Ayano et al., 2015; Gilfillan et al., 2008; Iguchi‐Manaka et al., 2008; Kearney, Ramsbottom, Voskoboinik, Darcy, & Oliaro, 2016). Previous studies examining the role of CD226 have primarily focused on the function of immune cells. It has recently become appreciated that CD226 might be linked to neuronal functions. For instance, CD226 is robustly expressed in the brain and specifically in the hilus of the dentate gyrus and stratum lucidum aligned along the pyramidal cells in the hippocampal CA3 area, which is the main brain region considered to be involved in the cognitive functions and anxiety‐like behaviors of mice. Moreover, CD226 could not be detected at its adult locations until postnatal day 12 (Zhang et al., 2009). Considering its notable expression pattern during development, we also found loss of CD226 did not show significant neuroanatomical alterations in the CD226KO mice (data not shown). Then we tested the effects of CD226 on spatial learning and memory. The results indicated that CD226 could enhance hippocampal synaptic plasticity using MWM test. In addition, we investigated the effect of CD226 deficiency on adaptive behaviors mediated by hippocampal neurons. The behavioral phenotypes of CD226KO mice in different experiments demonstrated that anxiety‐like behaviors could be genetically mapped through CD226. Interestingly, another cell adhesion molecule, the neuronal cell adhesion molecule (NCAM), also called CD56 is expressed in neural cells and immune cells. It is a prototypic marker of natural killer (NK) cells, and presents on a subset of CD4^+^ and CD8^+^ T cells. In nervous system, NCAM has been linked with serotonergic and dopaminergic neurotransmission. *NCAM* ^−/−^ mice showed increased anxiety‐like behavior and deficits in spatial learning and memory (Cremer et al., 1994; Stork et al., 1999).

While this study has shown new insights into the function of CD226, many questions are needed to be resolved. One limitation is that CD226KO mice used were not specific to neural cells. We cannot exclude the possibility that neuroinflammation mediated by CD226 plays a key role. Recently, we reported treatment targeting CD226 can ameliorate experimental autoimmune encephalomyelitis (EAE) by promoting IL‐10 expression via regulation of CD4^+^ T cell differentiation (Zhang et al., 2016). IL‐10 is an important modulator of inflammatory responses, and is critical for maintaining normal cognitive functions, especially learning and memory (Richwine, Sparkman, Dilger, Buchanan, & Johnson, 2009; Xiu et al., 2016). A striking feature of our study is that CD226 deficiency could increase IL‐10 expression in the hippocumpus. IL‐10 expression also trended toward increasement in cerebellum, but there was no statistical significance, and there was no significant difference in some inflammation cytokines (IFN‐γ and TNF‐α) expression (data not shown). Although many studies suggest that cytokines may influence synaptic plasticity and affect neurotransmitter metabolism in the CNS, especially serotonin levels and dopamine in the striatum (McAfoose & Baune, 2009; Miller, Maletic, & Raison, 2009), the mechanisms by which CD226 deficiency affects cognitive performance and anxiety are still unclear and need further investigation. On the other hand, no single mechanism is suggested to explain the complex role of interactions between CD226 and its ligands in CNS. It has been reported that nectin‐2 (CD112), one of ligands of CD226 has two forms of splicing variants, nectin‐2α and ‐2δ (Mizoguchi et al., 2002). Nectin‐2α was found to be expressed in both cultured mouse neurons and astrocytes. Interestingly, nectin‐2δ was only expressed in the astrocytes. Genetic ablation of nectin‐2 caused degeneration of astrocytic perivascular endfoot processes and neurons in the cerebral cortex (Miyata et al., 2016). In contrast, CD155, the other ligand of CD226 was not expressed in the adult central nervous system (CNS), but was expressed in glioblastoma (Enloe & Jay, 2011; Sloan et al., 2004). These clues together raise concern about potential effects of CD226 interaction with its ligands in brain.

In summary, CD226KO mice exhibited enhanced spatial learning and memory, and deletion of CD226 could also decrease the anxiety‐like behaviors. Together, these studies suggest that CD226 plays an important role in cognition and anxiety in mice. Moreover, the CD226 signaling pathway could potentially be considered as a novel candidate for intervention in mental disorders.

## CONFLICT OF INTEREST

The authors declare that there are none conflict of interest.
